# Discovery of Porcine microRNAs and Profiling from Skeletal Muscle Tissues during Development

**DOI:** 10.1371/journal.pone.0003225

**Published:** 2008-09-16

**Authors:** Ting-Hua Huang, Meng-Jin Zhu, Xin-Yun Li, Shu-Hong Zhao

**Affiliations:** Key Laboratory of Agricultural Animal Genetics, Breeding, and Reproduction of Ministry of Education & Key Laboratory of Swine Genetics and Breeding of Ministry of Agriculture, Huazhong Agricultural University, Wuhan, People's Republic of China; Fred Hutchinson Cancer Research Center, United States of America

## Abstract

MiRNAs (microRNAs) play critical roles in many important biological processes such as growth and development in mammals. In this study, we identified hundreds of porcine miRNA candidates through *in silico* prediction and analyzed their expression in developing skeletal muscle using microarray. Microarray screening using RNA samples prepared from a 33-day whole embryo and an extra embryo membrane validated 296 of the predicted candidates. Comparative expression profiling across samples of longissimus muscle collected from 33-day and 65-day post-gestation fetuses, as well as adult pigs, identified 140 differentially expressed miRNAs amongst the age groups investigated. The differentially expressed miRNAs showed seven distinctive types of expression patterns, suggesting possible involvement in certain biological processes. Five of the differentially expressed miRNAs were validated using real-time PCR. *In silico* analysis of the miRNA-mRNA interaction sites suggested that the potential mRNA targets of the differentially expressed miRNAs may play important roles in muscle growth and development.

## Introduction

The recent discovery of miRNAs introduced a new mechanism of gene expression regulation [1], [2]. Despite the fact that biological functions have been assigned to only a few dozen miRNAs, it is becoming apparent that miRNAs participate in the regulation of a variety of developmental and physiological processes [3]. Not surprisingly, recent studies have shown that miRNAs play important roles in the regulation of muscle development. The functional characterization of miR-1 and miR-133 has been an important step in our understanding of miRNA-mediated muscle development. miR-1-1 and miR-1-2 were first found to be specifically expressed in mouse cardiac and skeletal muscle precursor cells and were found to be transcriptionally regulated by the myogenic differentiation factors MyoD, Mef2, and SRF [4]. Overexpression of miRNA-1 in the mouse developing heart has a negative effect on muscle proliferation as it targets the transcription factor that promotes ventricular cardiomyocyte expansion, Hand2 [4]. In *Drosophila*, the expression of miR-1 is controlled by the Twist and Mef2 transcription factors [5]. Investigation of a loss-of-function phenotype of *Drosophila* miR-1 showed that miR-1 is not required for the formation or physiological function of the larval musculature, but is required for the post-mitotic growth of larval muscle [5]. Recent studies showed that miR-1 promotes myogenesis by targeting histone deacetylase 4 (HDAC4), a transcriptional repressor of muscle gene expression, and that miR-133 enhances myoblast proliferation by repressing serum response factor (SRF) [6], both examples of new molecular mechanisms to regulate skeletal muscle gene expression and embryonic developmental [6].

Another miRNA, miR-206, has also been characterized as a muscle regulator in recent studies. In co-operation with miR-133, miR-206 can repress myoblast fusion by targeting the connexin 43 (Cx43) gap junction channels without altering the Cx43 mRNA level [7]. These findings have generated more detailed insights into the mechanisms underlying the myogenesis process and have uncovered different pathways that lead to myofiber proliferation and differentiation. However, the complete roles of miRNAs in muscle growth & development still remain to be elucidated.

In mammals, muscle mass is mainly determined by the number and size of muscle fibers. In the pig, for example, the number of muscle fibers is prenatally determined during primary and secondary muscle fiber formation, while the postnatal hypertrophy process then increases the length and diameter of these fibers. Primary muscle fiber formation begins at approximately 30 days following gestation. Secondary muscle fiber formation begins at about 50 to 60 days post-gestation, when myoblasts align and fuse to form secondary muscle fibers at the surface of existing primary muscle fibers [4]. Identification of genes governing these processes will provide insights into the regulation of muscle growth. Currently, numerous genes, including growth factors, regulatory proteins, receptors, and transcription factors have been identified as participating in the regulation of the myogenesis. However, the underlying molecular pathway elements, such as the decisive secondary regulatory factors of the major genes responsible for controlling prenatal muscle growth, remains poorly understood.

We hypothesized that there were more miRNAs associated with muscle growth and development in prenatal pigs yet to be discovered. Profiling of transcriptome changes of mature miRNAs isolated from key developmental stages is a promising technique to use in uncovering these miRNAs. In the present study, we identify miRNAs whose expression has not previously been reported in pigs. Our results also identify a number of differentially expressed miRNAs that could represent new regulatory elements in muscle growth and development.

## Results and Discussion

### Identification of porcine miRNA candidates

#### *In silico* porcine miRNA prediction by homolog searches

We made use of the property of miRNAs to be highly conserved between closely related species in order to predict novel porcine miRNA candidates [8]. Pair-wise comparison of the porcine genomic sequences (August 2007) to hairpin sequences collected from mirBase (Version 10.0) resulted in 12,048 alignments. After removing the redundant alignments, we ended up with a total of 775 unique porcine miRNA candidates (Table S1). All candidates were found to have the potential for the hairpin-loop secondary structures typical to known miRNA transcripts. Among these candidates, 49 had been reported while the remaining were new.

Homolog search and *de novo* prediction are two typical approaches widely used in miRNA prediction. The homolog search approach is essential in our study since the porcine genome is not yet available for a direct prediction. Although the approach is limited by its inability to detect less conserved miRNAs, it is a nonetheless efficient and cost-effective.

#### Detection of expressed porcine miRNAs by microarray hybridization

To validate these miRNA candidates, a recently developed mammalian miRNA microarray was used to evaluate the expression of porcine miRNAs. At the design time of the microarray, there were 576 human miRNAs, 238 rat miRNAs and 358 mouse miRNAs reported. After removing the redundant sequences, there remained 743 unique mature miRNA sequences. The microarray was designed to contain 743 probes complementary to these sequences (See probe list of the microarray in Table S2). The *in silico* prediction mentioned above was based on the alignment of the reported miRNAs of human, mouse and rat to the porcine genomic sequences. As expected, the microarray covered all of the candidates found by this method, and thus can be used to detect their expression.

Microarray hybridization with RNA samples prepared from the 33-day post-gestation stage porcine whole embryo (E33.f) and placenta (P33.p) detected expression of 296 miRNAs (230 in E33.f and 275 in E33.p, signal>2Mean+2SD. See full list in Table S3.1). For the 49 porcine miRNAs deposited in miRbase, 41 of them were detected (35 in E33.f and 39 in E33.p). The six porcine miRNAs identified by Kim et al. were also detected [9]. The remaining 255 miRNAs have not been previously reported to be expressed in pig. We also found a large number of probes that showed strong signals but were not included in our candidate list, such as the miR-13 and miR-557. The failure to detect these candidates by the homolog search method is possibly due to the fact that only part of the porcine genome (60%) was available at the time.

The first reported porcine miRNA was the identification of the mir17-92 cluster using the homolog search method [10]. A more extensive homology search has since been performed by Kim et al. [9]. They identified 58 candidates and validated six of them by northern blot. Other miRNA entries in miRBase are predictions found by genomic comparisons with other model organisms such as human, mouse and rat without proof of expression [11]. There are 49 miRNAs reported so far. Our experiments expanded the number of porcine miRNAs (with identified sequence and confirmed expression) to 116 (Table 1 lists the top 20 highly expressed new miRNAs. See the full list in Table S4.1 and the predicted secondary structures in Table S4.2).

**Table 1:** New porcine miRNAs identified in 33 day post-gestation samples of whole embryo (E33.f) and placenta (E33.p).

MiRNA Name	Microarray Probe Sequence	Porcine Trace Sequence	Normalized Expression Level	
			E33.p	E33.f
ssc-let-7d	ACTATGCAACCTACTACCTCT	gnl|ti|1380820092	7274.33	25372.67
ssc-let-7e	ACTATACAACCTCCTACCTCA	gnl|ti|1577748346	16353.33	18411.33
ssc-mir-10b	ACAAATTCGGTTCTACAGGGTA	gnl|ti|2022730406	16918.67	7983.33
ssc-mir-124a-1	TGGCATTCACCGCGTGCCTTAA	gnl|ti|1420670121	21323	373
ssc-mir-15b	TGTAAACCATGATGTGCTGCTA	gnl|ti|2020963538	9361	12985
ssc-mir-16-1	CGCCAATATTTACGTGCTGCTA	gnl|ti|1579971821	22503.33	26688
ssc-mir-17	ACTACCTGCACTGTAAGCACTTTG	gnl|ti|1579909832	32828.67	9736.67
ssc-mir-191	AGCTGCTTTTGGGATTCCGTTG	gnl|ti|2025394035	9408.33	14004.33
ssc-mir-199(a/b)	AACCAATGTGCAGACTACTGTA	gnl|ti|(2019854499/1377265104)	40134.67	42470
ssc-mir-19b-1	TCAGTTTTGCATGGATTTGCACA	gnl|ti|1579909838	30333.33	8530
ssc-mir-22	ACAGTTCTTCAACTGGCAGCTT	gnl|ti|1377639070	5877.33	27348.67
ssc-mir-29a	AACCGATTTCAGATGGTGCTA	gnl|ti|860609555	5892.33	6809.67
ssc-mir-30b	AGCTGAGTGTAGGATGTTTACA	gnl|ti|1574275341	6958	17706.67
ssc-mir-30d	CTTCCAGTCGGGGATGTTTACA	gnl|ti|1008617003	14482.67	19839.33
ssc-mir-320	TTCGCCCTCTCAACCCAGCTTTT	gnl|ti|2027985691	9252.67	21585.67
ssc-mir-376a-1	ACGTGGATTTTCCTCTATGAT	gnl|ti|1008637782	11473	10293
ssc-mir-382	CGAATCCACCACGAACAACTTC	gnl|ti|775596795	3858.33	10386.33
ssc-mir-487b	AAGTGGATGACCCTGTACGATT	gnl|ti|851619303	7105.67	13520
ssc-mir-99a	CACAAGATCGGATCTACGGGTT	gnl|ti|2020960585	33488	35344
ssc-mir-185	GAACTGCCTTTCTCTCCA	gnl|ti|1575367821	6678.67	10275.67

### Global miRNA expression profiling of porcine skeletal muscle tissues

#### An overview of the expression profile

**Figure 1:**
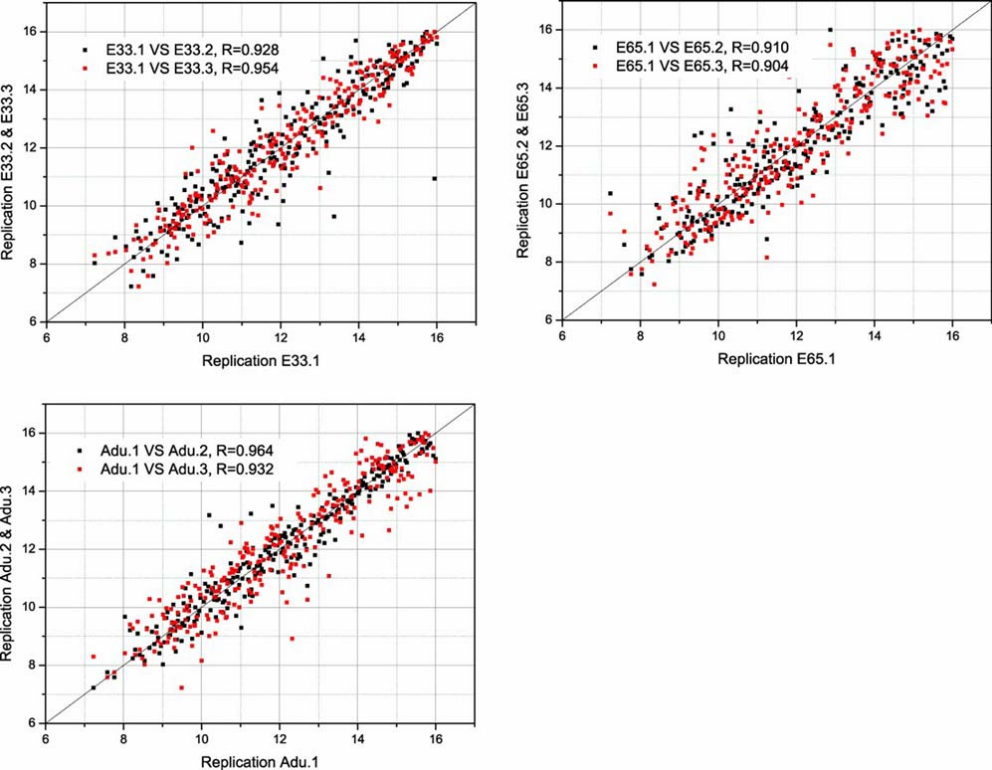
Reproducibility of the microarray experiments. We examined the miRNA expression in three developmental stages of skeletal muscle (E33, E65 and Adu). Samples from each stage were isolated in triplicate and hybridized to the microarray. Scatter plots demonstrate the pair-wise comparison between each two sets of triplicates. The R represents the Spearman correlation coefficient.

To identify the miRNAs that might be involved in muscle development and to discriminate these from the miRNAs possibly involved in promoting or repressing muscle myogenesis and differentiation, we carried out a comparative miRNA expression profile across skeletal muscle samples collected from pigs of 33-days post-gestation (E33), 65-days post-gestation (E65) and adult age (Adu). Samples from each age group were collected independently and the analysis performed in triplicate to ensure reliability. Comparisons between each of the replicates showed that the replicates have good reproducibility (Figure 1).

The use of short RNA probes antisense to the mature miRNA sequence has not proven to be an effective approach to reliably quantify the expression differences between miRNAs that have only one mismatch or a few mismatches [12]. Luo et al. previously performed a sensitivity test of the microarray using the artificially transcribed miRNA of let-7a to hybridize to the let-7 probe set (let-7a to let-7g, let7-i). Their results showed that the microarray utilized in this study was able to distinguish between the mismatched sequences, but was unable to distinguish between the highly similar sequences [13]. Therefore microarray results for closely related miRNAs should be interpreted with caution, as expression differences of a given miRNA could be exaggerated or diminished by the expression of their paralogs.

Of the 576 miRNAs on the microarray, 256 (44%) were expressed in the muscle samples. Of those expressed, 227 were in E33 and 228 in E65, while only 163 were expressed in Adu (see Table S3.2). Taking into account the fact that miRNAs are negative regulators of coding genes that act by either inhibiting translation or inducing mRNA degradation of the target gene [3], [14], [15], these results suggest lower expression levels of the coding genes regulated by the miRNAs in the prenatal stages. The modulation of muscle development processes is triggered by sequential events of gene activation and inhibition. The differences in miRNA expression between the ages detected in this study support the complexity of their roles in muscle development.

#### Differentially expressed miRNAs detected by the microarray

Of the 256 miRNAs detected by the microarray, expression levels of 140 of them changed significantly between the developmental stages investigated (Fold change>2, p<0.001, FDR<0.001, see Table S5) and 51 changed more than ten-fold (Table 2). For example, the average increase of miR-486 signal from E33 to E65 was 3.3-fold, and 13.4-fold from E65 to Adu; the average increase of miR-376b signal from E33 to E65 was 4.6-fold, but decreased 54.7-fold from E65 to Adu, and therefore in Adu it appeared 11.9-fold lower than in E33; miR-422a signal increased more than 6.9-fold from E33 to E65, after which it remained stable; miR-495 signal was strong in E33 and E65, but nearly undetectable in the Adu stage. Interestingly, we found that three miRNAs (miR-363, miR-365 and miR-422b) were differentially expressed between E33 and Adu, despite their expression not being significantly different when comparing either E33 to E65 or E65 to Adu. This may represent a type of long term regulation.

**Table 2:** MiRNAs differentially expressed between E33, E65 and Adu stages (Fold change >10.0, p<0.001 and FDR<0.001).

MiRNA Name	E65/E33	Adu/E65	Adu/E33	MiRNA Name	E65/E33	Adu/E65	Adu/E33
miR-214	-	0.13	0.06	miR-493	4.62	0.08	-
miR-422a	6.93	-	15.01	miR-409-5p	2.35	0.08	0.19
miR-503	-	0.12	0.07	miR-379	-	0.09	0.21
miR-497	-	5.80	11.09	miR-95	-	13.23	16.38
miR-721	-	5.00	11.07	miR-369-5p	2.85	0.06	0.16
miR-189	-	7.79	10.37	miR-557	-	30.97	23.00
miR-378	-	6.24	11.58	miR-655	-	0.06	-
miR-487a	2.92	5.34	15.59	miR-656	-	0.08	-
miR-680	-	4.98	14.45	miR-182	0.06	-	0.13
miR-127	-	0.03	0.04	miR-376a	4.65	0.06	0.26
miR-495	-	0.01	0.02	miR-365	-	-	14.39
miR-411	-	0.01	0.04	miR-486	3.26	13.44	43.75
miR-487b	4.41	0.04	-	miR-323	-	0.06	0.09
miR-29a	1.92	16.78	32.24	miR-660	2.27	0.08	0.17
miR-193b	-	22.41	22.68	miR-409-3p	-	0.06	-
miR-29b	-	15.49	32.19	MIR-202	3.74	0.03	0.13
miR-376b	4.59	0.02	0.08	miR-382	-	0.03	0.11
miR-29c	-	17.44	17.45	miR-503	-	0.09	0.07
miR-376a	3.20	0.03	0.10	miR-431	3.90	0.04	0.15
miR-335	-	0.05	0.08	miR-410	-	0.09	-
miR-411	3.28	0.03	0.09	miR-150	-	20.74	48.39
miR-532	-	0.05	-	miR-380-3p	-	0.06	0.19
miR-299-5p	4.73	0.05	-	miR-432	3.70	0.07	-
miR-362	-	0.09	0.13	miR-196a	0.07	6.10	-
miR-455-3p	-	0.08	-	miR-329	6.35	0.03	0.19

Pair-wise comparisons showed that large numbers of miRNAs are differentially expressed between any given two ages. In addition, the number of differentially expressed miRNAs as well as the value of the average fold changes varied between the three developmental ages investigated. As shown in Table 3, the number of differentially expressed miRNAs between E33 and E65 is much smaller than between E65 and Adu, and the value of the average fold change between E33 and Adu is much lower than between E65 and Adu. These findings show that the expression patterns of the three ages are unique.

**Table 3:** Summarization of differentially expressed miRNAs.

Comparison Groups	Change Direction	Number of Regulated miRNAs			Average FC (fold change)
		>2-fold	>5-fold	>10-fold	
E33 VS E65	up	28	3	0	4
	down	25	9	2	5
	all	53	12	2	4
E65 VS Adu	up	60	25	8	6
	down	50	40	31	18
	all	110	65	39	12
E33 VS Adu	up	51	27	16	10
	down	50	29	12	8
	all	101	56	28	9

Of the three miRNAs reported as regulators of development in skeletal and cardiac muscle, miR-206 was found to be up-regulated 2.9-fold in Adu compared to E65, but the expression variance of miR-1 and miR-133 failed to reach statistically significant levels. These two miRNAs showed a high level of expression in the microarray analysis, thus technical error could be ruled out. It should be noted that the functional discovery of these miRNAs was made mostly in cell culture systems, which may differ from the in vivo system.

Several of the differentially expressed miRNAs identified here were shown to play roles in growth and development related processes in recent studies. These include miR-214, miR-140, miR-150, miR-10, as well as miR-181. In the zebrafish, miR-214 can modulate hedgehog signaling, thus changing muscle cell fate [18], and miR-10 was shown to represses HoxB1a and HoxB3a, which are involved in patterning the anterior-posterior axis [19]. In mouse cells, the cartilage specific miRNA, miR-140, targets the histone deacetylase 4 (HDAC4), suggestive of a role in long bone development [20]. In mature B and T cells, the miR-150 was found to block early B cell development when expressed prematurely, and also found to control B cell differentiation by targeting the transcription factor of c-Myb [21]. Furthermore, miR-181 was found to be involved in the process of mammalian skeletal-muscle differentiation, by targeting the homeobox protein Hox-A11 during mammalian myoblast differentiation [22]. These findings suggest that identifying differentially expressed miRNAs may lead to the discovery of miRNAs related to muscle growth and development.

#### Validation of the microarray results by direct quantification

**Figure 2:**
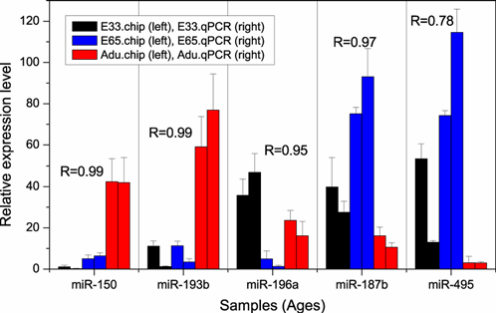
Validation of the microarray results using Real-time PCR method. Expression levels of five miRNAs (miR-150, miR-193b, miR-196a, miR-187b and miR-495) were detected by Real time PCR (right) and microarray (left). We have made a scaling to the raw data of Real time PCR to make it comparable to the microarray data. R represents the Pearson correlation coefficient.

Five representative differentially expressed miRNAs (miR-150, miR-193b, miR-196a, miR-187b and miR-495) were chosen for validation by the stem–loop RT–PCR based real-time PCR method [6] using three independent samples (The primer sequences are available in Table S6). The expression levels for miR-150, miR-193b, miR-187b and miR-196a, as determined by RT-PCR, were in concordance with the normalized microarray data (Pearson correlation coefficient >0.9, q value<0.001, Figure 2). In general, the results of qPCR validated the microarray results. An exception was miR-495, for which the expression levels in E33 and E65 varied dramatically. Although we have not verified the exact cause, the variance may come from biological differences between the samples. Furthermore, it should be noted that the purification process of the stem–loop RT–PCR assay is unable to completely remove long RNA nucleotides, thus we cannot exclude the possibility that the precursors are also quantified [6].

#### Distinctive miRNA expression patterns during muscle development

**Figure 3:**
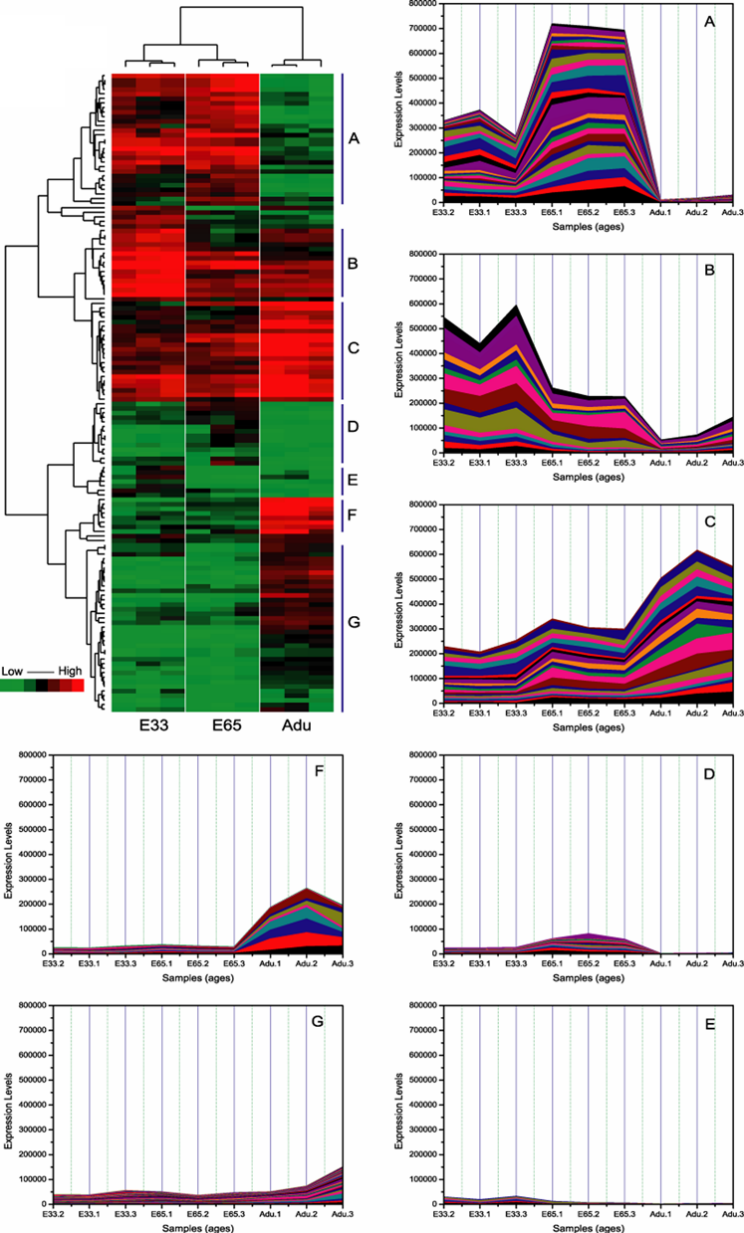
Hierarchical cluster analysis. We performed a data adjustment (median center and normalization) in the cluster analysis. The color codes of red, white, black and dark green represents expression levels of high, average, low and absent respectively. A detailed view of the miRNA expression levels in clustering patterns is shown in the plot areas from A to G.

To visually illustrate the expression type of the miRNAs being expressed during different developmental stages, a hierarchical cluster analysis was performed for the differentially expressed miRNAs. The results show that the miRNA expression patterns fall into seven typical categories: A) prenatally expressed, expression level increased between E33 and E65; B) universally expressed, expression level decreased between E33 and E65; C) universally expressed, expression level increased through the three ages; D) moderately expressed in E65, expression levels in E33 and Adu nearly undetectable; E) moderately expressed in E33, expression levels in E65 and Adu nearly undetectable; F) postnatally expressed, expression nearly undetectable in prenatal ages; G) moderately expressed, expression level increase through the three ages. The expression patterns described above are clearly reflected by the formation of several big clusters in the tree map of the clustering results (Figure 3).

The myogenesis process takes place mostly in the prenatal stage and becomes almost inhibited in the postnatal stage [16]. It has been demonstrated in the pig that primary muscle fiber formation begins at approximately 30 days post-gestation and the secondary muscle fiber formation begins at about 50 to 60 days post-gestation [17]. The categories of miRNA expression patterns described above provide a sensible basis for generating specific hypotheses of how miRNAs function in the biological context of the developmental ages investigated. The prenatally expressed miRNA clusters may include miRNAs that play roles in the promotion of myogenesis (Figure 3 A and B). In contrast, the postnatally expressed miRNAs clusters may include miRNAs that act as inhibitors of myogenesis (Figure 3 C and F). The E33 and E65 highly expressed miRNA clusters may include miRNAs that play roles in the process of primary and secondary muscle fiber formation, respectively (Figure 3 E and D). In previous studies, we found that although the secondary muscle fiber formation took place later than the primary muscle fiber formation process, the two temporally overlapped at the beginning of secondary muscle fiber formation [17]. This may be the primary reason why a large number of miRNAs are expressed both at the E33 stage and the E65 stage (Figure 3 A, B and F).

#### Differentially expressed miRNAs may play important roles in porcine muscular development

A major challenge to understanding the function of these developmentally regulated miRNAs is the question of target identification. It is commonly recognized that the miRNA and its targets must be located in the same subcellular position to make the biological interactions operable, thus the spatial and temporal information of miRNA expression may narrow the search for miRNA target candidates.

The differentially expressed miRNAs detected by this microarray analysis provide a valuable candidate list of muscle growth and development related miRNAs. In this analysis, we used a well established miRNA-target dataset generated by TargetScan to investigate the possible functions of these miRNAs and to provide evidence for their involvement in the muscle development process [23], [24]. The relevant mRNA sequences used in this analysis are orthologous genes. In addition, the target sites were characterized as evolutionarily conserved in five species (human, mouse, rat, dog and chicken), a criterion that also acted as a good filter for false positive assignments of miRNAs to genes [24], [25]. Altogether we analyzed 6,654 genes that have at least one predicted miRNA binding site in their 3′ UTR, and a total of 84 miRNA families in the TargetScan datasets. As a result, we obtained a total of 24,555 predicted miRNA-mRNA interaction sites (Table S7). As we expected, most of the miRNAs investigated targeted hundreds of genes and over 65% of the targets were regulated by more than one miRNA (Tables S8.1 and S8.2). The high degree of connectivity between the miRNA-mRNA pairs supports the idea that these miRNAs have extensive and complicated roles during the muscle development process. Three genes, NFIB, TNRC6B and ZNF148 assigned the highest number of miRNA interaction sites. The NFIB gene was previously identified as an activator of the differentiation-specific genes, such as MCSFR [26]. TNRC6B was co-purified with a cytoplasmic HeLa cell protein complex containing AGO2, DICER, and MOV10, and thus is implicated in mediating miRNA-guided mRNA cleavage in HeLa cells [27]. ZNF148 (alias ZBP89) was originally reported as a gastrin gene expression repressor [28], [29] and recently, studies of mice expressing only ZBP89-delta-N showed significant growth delay and a reduction of viability [30]. GO terms and KEGG pathway annotation of the miRNA targets using the DAVID gene annotation tool (http://david.abcc.ncifcrf.gov/) further illustrate the possible roles and mechanisms of these differentially expressed miRNAs in muscle development (Document S1).

The above analyses provide an overview investigation on the possible functions of differentially expressed miRNAs based on computationally predicted target datasets. Although the accuracy of the computational approaches for identification of mammalian miRNA targets is still limited [33], these results will definitely advance the hypothesis-driven functional studies of these miRNAs.

## Materials and Methods

### Homolog search for miRNA candidates

Analysis of the current porcine genomic draft sequences (August 2007) was performed by comparing porcine genomic sequences with both experimentally confirmed and predicted data sets from other species using BLAST (Basic Local Alignment Search Tool). The alignments, requiring at least 90% pre-miRNA similarity and 100% mature miRNA similarity, were reserved for further study. The predicted miRNA secondary structure was generated by the RNAfold software package (http://www.tbi.univie.ac.at/ivo/RNA/RNAfold.html). We also checked the phylogenetic shadowing profile of these sequence pairs as characterized by the miRNA coding arm, which suffered the highest selective pressure, and then in succession the non-coding arm, the stem region, the loop region, and the flanking sequence. The candidates not following these rules were removed from the datasets. After these steps, we ended up with hundreds of miRNA candidates.

### Samples and RNA preparation

Our experiments included three RNA samples isolated from three independent fetal or adult pigs. Sample collection was approved by the ethics committee of Huazhong agricultural university. The longissimus tissues were dissected after removing the epimysium coverings. These samples were snap-frozen in liquid nitrogen and stored at −80°C. Total RNA was isolated using a Trizol protocol (Invitrogen). After quantification, the RNA was isolated using PEG (polyethylene glycol) and labeled by RNA ligase according to the method of Thomson et al. [34].

### Microarray hybridization and data analysis

The microarrays used in this study were bought from CapitalBio Company (NO. 225011). The hybridization was done by the CapitalBio Company service. In brief, labeled RNA was dissolved in 16 ul hybridization mixture (15% formamide; 0.2% SDS; 3×SSC; 50×Denhardt's) and hybridized overnight. The slides were washed in 0.2% SDS, 2×SSC for four minutes at 42°C, and in 0.2×SSC for four minutes. The slides were scanned using the LuxScan 10 K/A scanner (CapitalBio Company) and the raw pixel intensities were extracted using the LuxScan3.0 software (CapitalBio Company). The median pixel intensities were background subtracted. Hybridization signals that failed to exceed the average background value by more than two standard deviations (Signal>Mean+2SD) were excluded from analysis. In all of the three duplicate slides, probe signal>Mean+2SD was classified as detected (for E33.p and E33.f, no duplicate experiments were performed, thus signal exceeding 2×Mean+2SD were defined as detected.). The data were normalized between slides from different ages groups using the quantile normalization method proposed by Bolstad et al [35]. The differentially expressed genes, classified as those with Fold changes>2, P value<0.001 and FDR<0.001, were selected using the SAM software, version 2.1 (Significance Analysis of Microarrays, http://www-stat.stanford.edu/tibs/SAM/). The subsequent analysis of miRNA targets prediction and target gene functional annotation was performed using the TargetScan software (http://www.targetscan.org/) and the DAVID gene annotation tool (http://david.abcc.ncifcrf.gov/), respectively.

### Stem-Loop Real-time RT-PCR

A miRNA quantification method similar to that described by Chen et al. [6] was used to validate the microarray data. Three independent samples from each time point were analyzed. In brief, the assay was performed using Stem–loop RT followed by SYBR Green Real-time PCR analysis. Firstly, 1 µg total RNA was reverse transcribed using 200 U M-MLV Reverse Transcriptase (Takara: 02640A) and 1 µl Stem-loop RT primer in an Applied Biosystems 9700 Thermocycler with incubation at 30°C for 15 min, 42°C for 60 min and 85°C for 5 min. Importantly, all reverse transcriptase reactions were run along with “no-template controls”. The no-template controls gave non-detectable signals in all samples, confirming the high specificity of the miRNA quantification assay. Real-time PCR was performed using a standard SYBR Green PCR kit (Toyobo: QPK-201) on the BIO-RAD iQ5 Real-Time PCR Detection System. Porcine Met-tRNA was used as an internal control and all reactions were run in triplicate. The ΔΔCt method was used to determine the expression level differences between surveyed stages [36]. The significant level was set to 0.05.

## Supporting Information

Document S1 (0.08 MB DOC)

Table S1 (0.16 MB XLS)

Table S2 (0.12 MB XLS)

Table S3 (0.19 MB XLS)

Table S4 (0.08 MB XLS)

Table S5 (0.04 MB XLS)

Table S6 (0.02 MB XLS)

Table S7 (1.87 MB XLS)

Table S8 (0.51 MB XLS)
