# Supplementary material for: Discovery of Porcine microRNAs and Profiling from Skeletal Muscle Tissues during Development
Source: PLoS One. 2008 Sep 16;3(9):e3225. doi: 10.1371/journal.pone.0003225 (PMC2528944; doi:10.1371/journal.pone.0003225)
Supplement: Document S1 — (0.08 MB DOC) [file pone.0003225.s001.doc]

**GO terms and KEGG pathway annotation of the miRNA targets**

To fully inspect the function of the differentially expressed miRNAs, we collected the top 25 percent of the predicted miRNA targets that have been assigned the highest numbers of miRNAs and performed a GO term and KEGG pathway annotation using the DAVID gene annotation tool (H[TUhttp://david.abcc.ncifcrf.gov/UT](http://david.abcc.ncifcrf.gov/)H). GO term annotation results showed that cellular physiological process, metabolism, regulation of cellular process and regulation of physiological process are the most significantly enriched GO terms. Interestingly, the growth & development related GO terms (system development, morphogenesis, organ development, cell differentiation, cell growth, regulation of growth, embryonic development, regulation of development and pattern specification) represented up to 45% (9/20) of the significantly enriched GO terms and ~14% of the target genes analyzed. This suggests that the muscle developmental patterning of the three investigated stages is subject to regulation by the miRNAs and miRNA targets (Table 1).

The regulator pathway annotation was performed based on scoring and visualization of the pathways collected in the KEGG database (H[TUhttp://www.genome.jp/kegg/UT](http://www.genome.jp/kegg/)H). From this analysis, 22 pathways were over-represented, suggesting that these pathways are significantly regulated in the three stages of muscle development investigated in this study (Table 2). Interestingly, most of the pathways have been shown to be involved in the growth & development process, including the skeletal muscle development process. For example, in the top four enriched pathways, the MAPK pathway can regulate a wide variety of cellular functions, including cell proliferation, differentiation, and stress responses [1]. The axon guidance pathway, the process by which H[neurons](http://en.wikipedia.org/wiki/Neuron)H send out H[axons](http://en.wikipedia.org/wiki/Axon)H to reach the correct targets, was found as a subfield of H[neural development](http://en.wikipedia.org/wiki/Neural_development)[2]. Signaling from the Wnt pathway has been found to play a central role in controlling embryonic development in organisms ranging from hydra to human, and recent studies show that WNT signaling induces myoblast differentiation in the limb [2]. Altogether, these pathway analysis results further illustrated the possible roles and mechanisms of these differentially expressed miRNAs in muscle development.

Table 1. GO Functional Enrichment of the miRNA Targets (DAVID, grouped according to

biological process of level 2)

| GO Term | Count | % | P-Value |
| --- | --- | --- | --- |
| cellular physiological process | 828 | 34.29% | 1.16E-15 |
| metabolism | 644 | 26.67% | 2.68E-07 |
| regulation of cellular process | 412 | 17.06% | 3.44E-38 |
| regulation of physiological process | 404 | 16.73% | 5.66E-37 |
| cell communication | 287 | 11.88% | 3.32E-05 |
| negative regulation of biological process | 106 | 4.39% | 6.88E-13 |
| system development | 90 | 3.73% | 2.46E-19 |
| cell adhesion | 74 | 3.06% | 3.19E-05 |
| morphogenesis | 71 | 2.94% | 2.91E-06 |
| organ development | 69 | 2.86% | 1.75E-07 |
| cell differentiation | 66 | 2.73% | 2.62E-07 |
| positive regulation of biological process | 66 | 2.73% | 2.94E-04 |
| death | 49 | 2.03% | 0.080669 |
| locomotion | 26 | 1.08% | 0.016152 |
| cell growth | 18 | 0.75% | 0.032177 |
| regulation of growth | 15 | 0.62% | 0.053749 |
| embryonic development | 14 | 0.58% | 0.002142 |
| regulation of development | 11 | 0.46% | 0.04342 |
| regulation of gene expression, epigenetic | 7 | 0.29% | 0.074249 |
| pattern specification | 6 | 0.25% | 0.029909 |

Table 2. KEGG pathway annotation of the miRNA Targets

| Kegg Pathway | Count | % | P-Value |
| --- | --- | --- | --- |
| hsa04010:mapk signaling pathway | 42 | 1.74% | 1.21E-06 |
| hsa04360:axon guidance | 36 | 1.49% | 6.04E-12 |
| hsa04510:focal adhesion | 30 | 1.24% | 3.22E-04 |
| hsa04310:wnt signaling pathway | 28 | 1.16% | 2.64E-06 |
| hsa04810:regulation of actin cytoskeleton | 25 | 1.04% | 0.009520299 |
| hsa04020:calcium signaling pathway | 23 | 0.95% | 0.004948315 |
| hsa04910:insulin signaling pathway | 20 | 0.83% | 0.003191896 |
| hsa04720:long-term potentiation | 20 | 0.83% | 2.28E-08 |
| hsa04350:tgf-beta signaling pathway | 20 | 0.83% | 3.55E-06 |
| hsa04530:tight junction | 17 | 0.70% | 0.009263114 |
| hsa04520:adherens junction | 17 | 0.70% | 7.81E-05 |
| hsa04540:gap junction | 16 | 0.66% | 0.002240164 |
| hsa04514:cell adhesion molecules (cams) | 15 | 0.62% | 0.06036325 |
| hsa04660:t cell receptor signaling pathway | 14 | 0.58% | 0.014374753 |
| hsa04070:phosphatidylinositol signaling system | 13 | 0.54% | 0.034658858 |
| hsa04730:long-term depression | 11 | 0.46% | 0.036309684 |
| hsa00562:inositol phosphate metabolism | 10 | 0.41% | 0.056196397 |
| hsa04120:ubiquitin mediated proteolysis | 10 | 0.41% | 0.002949404 |
| hsa04662:b cell receptor signaling pathway | 9 | 0.37% | 0.098331842 |
| hsa04320:dorso-ventral axis formation | 7 | 0.29% | 0.017029321 |
| hsa05120:epithelial cell signaling in helicobacter pylori infection | 7 | 0.29% | 0.092375613 |
| hsa01510:neurodegenerative disorders | 6 | 0.25% | 0.08831598 |

1. Tanoue T, Nishida E (2002) Docking interactions in the mitogen-activated protein kinase cascades. Pharmacol Ther 93: 193-202.

2. Huber AB, Kolodkin AL, Ginty DD, Cloutier JF (2003) Signaling at the growth cone: ligand-receptor complexes and the control of axon growth and guidance. Annu Rev Neurosci 26: 509-563.
